# First Screening of Entomopathogenic Nematodes and Fungus as Biocontrol Agents against an Emerging Pest of Sugarcane, *Cacosceles newmannii* (Coleoptera: Cerambycidae)

**DOI:** 10.3390/insects10040117

**Published:** 2019-04-25

**Authors:** Marion Javal, John S. Terblanche, Desmond E. Conlong, Antoinette P. Malan

**Affiliations:** 1 Department of Conservation Ecology and Entomology, Faculty of AgriSciences, Stellenbosch University, Private Bag X1, Matieland 7602, South Africa; jst@sun.ac.za (J.S.T.); des.conlong@sugar.org.za (D.E.C.); apm@sun.ac.za (A.P.M.); 2 South African Sugarcane Research Institute, 170 Flanders Drive, Mount Edgecombe, KwaZulu-Natal 4300, South Africa

**Keywords:** *Cacosceles newmannii*, *Metarhizium pinghaense*, sugarcane, entomopathogens, EPN, EPF, *Xenorhabdus khoisanae*

## Abstract

*Cacosceles newmannii* (Coleoptera: Cerambycidae) is an emerging pest of sugarcane in South Africa. The larvae of this cerambycid beetle live within the sugarcane stalk and drill galleries that considerably reduce sugar production. To provide an alternative to chemical control, entomopathogenic nematodes and fungus were investigated as potential biological control agents to be used in an integrated pest management system. The nematodes *Steinernema yirgalemense*, *S. jeffreyense*, *Heterorhabditis indica*, and different concentrations of the fungus *Metarhizium* *pinghaense* were screened for efficacy (i.e., mortality rate) against larvae of *C. newmannii*. The different biocontrol agents used, revealed a low level of pathogenicity to *C. newmannii* larvae, when compared to control treatments.

## 1. Introduction

The longhorned beetle *Cacosceles newmannii* Thompson 1877 is a cerambycid native to Mozambique, eSwatini and South Africa. The biology of this beetle is poorly known, and its host plants have not yet been fully determined, but might include species from the Myrtaceae family [1].

*Cacosceles newmannii* larvae were found in 2015 feeding on commercially grown sugarcane in the KwaZulu-Natal province of South Africa. Larvae dig galleries into the sugarcane stool and upwards from 8 to 20 cm into the bottom section of the stalk, but are most of the time found in the below-ground section of the sugarcane stalks, in the stool [2]. The reasons underlying the host shift of *C. newmannii* onto sugarcane remain unclear [3]. Regardless of the factor(s) determining the switch to sugarcane, this species has the potential to spread and cause considerable losses.

Cerambycids attacking sugarcane can become serious pests and have severe economic impact worldwide [4,5,6]. In Thailand, for instance, *Dorysthenes buqueti* (Guérin-Méneville 1844) (Coleoptera: Cerambycidae) populations increased 10-fold within a year, along with resultant damage observed in the affected sugarcane [7]. 

Biocontrol includes all plant protection methods that use natural mechanisms, such as parasitism, pathogenicity or predation, and help to complement or reduce the use of chemical insecticide applications in the crop [8]. Biocontrol strategies in integrated pest management are of growing interest in the context of recognized detrimental effects of chemical compounds on ecosystems and human health [9,10]. Among the various biocontrol agents commonly used, entomopathogenic nematodes (EPNs) have a relatively broad natural host range [11], and have shown their efficiency against many insect species [12] including soil-associated insects [13]. EPNs of the families Steinernematidae and Heterorhabditidae more specifically, occur naturally in soils throughout the world [14,15]. The free-living infective juveniles (IJs) actively move in the soil to find their hosts [16], making them a promising biocontrol agent for soil-associated insects [13] such as *C. newmannii*. The IJs enter their host through natural openings and in some cases through the cuticle [17]. They then release the symbiotic bacteria they carry in their gut, which rapidly cause the death of the insect [18]. The IJs develop into adults in the haemocoel of the infected insect, feeding on the bacteria. They develop and reproduce within the dead host until all the nutrients have been consumed, and a new cohort of IJs exits the dead insect in search of new hosts [19].

Fungi have also proven their efficacy in controlling sugarcane stem borers [20,21] as well as soil-inhabiting insects [22] including Coleoptera [23,24]. Infection by entomopathogenic fungi (EPF) requires the penetration of conidia into the insect’s body and their germination on the insect’s cuticle. They are then able to produce lethal toxins that ultimately kill the insects. Unlike nematodes, that are able to seek out their hosts, the conidia are motionless and are spread from the dead insect through air or water [25].

Given the potential for EPNs as biocontrol agents of insects, their effect against insect pests have been widely investigated [12,26], and multiple species have been commercialised worldwide [27]. However, they are globally rather infrequently used, especially in Africa [15], despite the large number of programs dealing with Coleoptera species [23]. Similarly, the increase in understanding of the fungal taxonomy helped develop the use of EPF. In South Africa specifically, the development of entomopathogenic fungi as biocontrol agents has shown an important growth over recent years [28]. However, EPF’s success as biocontrol agents is based on a strong investment in research and development, which limits their generalization [25]. Both EPN and EFP can be used as part of integrated pest management programs (IPM), together with other pest control tactics [29].

In this study, we tested the virulence of different locally isolated EPN species and an EPF on *C. newmannii* larvae.

## 2. Materials and Methods 

### 2.1. Source of Insects

*Cacosceles newmannii* larvae were collected by hand on sugarcane farms in the Entumeni District (28°55′S; 31°19′E) close to Eshowe, Kwazulu-Natal, South Africa [2]. They were kept in trays with peat and pieces of sugarcane provided as food before the experiments started. Larvae were weighed (Table 1) and cleaned with tap water and blotted dry before each experiment. Specimens are relatively difficult to find *en masse* in the field; therefore, most experiments could not be replicated.

For the experiments 1, 3, and 4 (see below), *Galleria mellonella* Linnaeus 1758 (greater wax moth, Lepidoptera: Pyralidae) larvae were used as a positive control, since they are known to be highly susceptible to the EPN used in this study [30]. For the experiment 5, *Cydia pomonella* Linnaeus 1758 (codling moth, Lepidoptera: Tortricidae) larvae were used as control. *Cydia pomonella* has been shown to be highly susceptible to several *Metarhizium* species [31].

### 2.2. Entomopathogenic Nematode 

We assessed the virulence of three difference laboratory reared nematode species, *Steinernema yirgalemense* Nguyen, Tesfamariam, Gozel, Gaugler & Adams 2004, *Steinernema jeffreyense* Malan, Knoetze & Tiedt 2015, and *Heterorhabditis indica* Poinar, Karunakar & David 1992, obtained from the EPN collection of the nematology laboratory at the Department of Conservation Ecology and Entomology, Stellenbosch University, South Africa. The three nematode species used naturally occur in South Africa [32], and are listed in Table 2.

#### 2.2.1. Experiment 1: EPN Virulence

Each of the three nematode species was inoculated to 10 *C. newmannii* larvae per treatment, along with 10 *G. mellonella* larvae used as a positive control. After weighing, each larva was placed individually in a 9 cm diam. Petri dish, to which a filter paper disk, moistened by adding 350 µL of distilled water, was added. Fifty µL containing 400 infective juveniles (IJ) were then pipetted on the filter paper of each of the 10 Petri dishes, for each nematode treatment (*n* = 30) [36]. Ten additional *C. newmannii* larvae were used as a negative control and received only 400 µL of water. The lid of each Petri dish was sealed with Parafilm (Pechiney Plastic Packaging, Neenah, WI, USA) to prevent the escape of insects. Petri dishes were then placed in a plastic container (one container per treatment), lined with wet paper towels to ensure air high humidity, and kept in an incubator at 25 °C. Generally, entomopathogenic nematodes are able to penetrate their host within 12 to 24 h [36]. However, larval mortality was checked every day for 3 days. Cause of mortality was confirmed by dissecting the dead larvae, with the aid of a light microscope, and visually determining the presence of nematodes inside the larvae. The experiment was conducted twice (trials 1 and 2) in order to confirm results. The total numbers of dead larvae per nematode species were then compared using a Pearson’s Chi-squared test in R (version 3.3.0 [37]). In all cases, we used a significance level of 5%.

#### 2.2.2. Experiment 2: Nematode Penetration

In order to confirm that nematodes were capable of effectively penetrating and entering the body of the insect, five *C. newmannii* larvae were inoculated again with a high number of nematodes (1000 IJs per larva), following the same protocol as described above, and kept at 25 °C for 2 days before being killed and dissected. Before dissection the larvae were washed with water to remove nematodes from the surface of the insects. The number of nematodes per insect was then scored with the aid of a light microscope, and the penetration value was computed according to the following formula [36]:

$$P=\frac{N\times 100}{T}$$

*P* being the penetration value, *N*, the average number of nematodes found in each larva and *T* the initial number of nematodes inoculated per larva. The three values of *P* were then statistically compared using a one sample t-test in R.

#### 2.2.3. Experiment 3: Nematode Development in Haemolymph

*Steinernema jeffreyense* was the only nematode species causing mortality of a *C. newmannii* larva (see Results). However, given the low pathogenicity level observed, additional experiments were performed to aid the understanding of the potential mechanisms involved in insect resistance. Ten *C. newmannii* were washed with distilled water, dried and perforated with a sterile insulin needle. A droplet of haemolymph was collected in a Petri dish, and immediately inoculated with *S. jeffreyense* IJs. The same protocol was repeated using 10 *G. mellonella* larvae as a control. Between 7 and 14 IJs were inoculated per droplet of haemolymph. The Petri dishes were then sealed with Parafilm, placed in a plastic container with wet paper towels and kept in an incubator at 25 °C. The numbers of males, females with and without eggs, IJs, and dead nematodes were checked after 48 h. Then, only the presence of progeny, and of fertilized females was recorded 72, 96 and 120 h after inoculation.

#### 2.2.4. Experiment 4: Virulence of Mutualistic Bacteria

Last, we tested whether the low level of pathogenicity of *S. jeffreyense* could be due to the inability of the nematode’s symbiotic bacteria, *Xenorhabdus khoisanae* Ferreira, Van Reenen, Endo, Sproër, Malan & Dicks 2013 [38], to grow in the insect’s haemolymph. The bacteria cell concentration of 1 × 10^7^ cells/ml was determined by counting the cells using a haemocytometer [39]. Larvae were first washed with pure ethanol. We then injected 2 µL of a suspension of bacteria in tryptic soy broth into *C. newmannii* larvae using sterile insulin needles, and volumes between 1 and 10 µL were injected in *G. mellonella* larvae as a positive control. In addition, 2 µL of distilled water was injected in five *C. newmannii* larvae as a negative control. Mortality was checked every day for 5 days. All insects injected with bacteria were dissected after death.

### 2.3. Experiment 5: Virulence of EPF 

We assessed the virulence of *Metarhizium pinghaense* Chen & Guo 1986 (Ascomycota: Hypocreales: Clavicipitaceae) at two different concentrations (1 × 10^7^ and 2 × 10^7^ spores per mL). *Metarhizium pinghaense* (previously identified as *M. anisopliae*) has been successfully commercialised in other countries [40,41]. The South African strain of *M. pinghaense* used in this study was isolated from an apple orchard and showed to be effective against codling moth and woolly apple aphid [42].

The conidia were freshly produced on Sabouraud dextrose agar (SDA) plates. Preliminary germination tests of freshly cultured conidia of *M. pinghaense* showed >90% germination of conidia in all cases. In order to determine the conidial concentration, 0.05% Tween 20 was added as a standard, to be able to get the conidia into suspension.

After weighing, *C. newmannii* larvae were dipped in a suspension of the fungal isolates (*n* = 10 larvae per concentration) for 15 sec, and placed in an empty petri dish for about 5 min in order to dry. Larvae were then covered by autoclaved peat, and distilled water was added to provide enough moisture for the larvae and the fungus to survive. Five *C. newmannii* larvae were used as a negative control and were dipped in water for 15 seconds, before being covered with wet peat. Ten *C. pomonella* larvae were used as a positive control for each concentration. 

Petri dishes were placed in an incubator at 25 °C, and larval mortality was then checked every week for three weeks. Cause of mortality was confirmed visually by the presence mycosis on the dead larvae, and the mortality levels observed after 21 days were statistically compared using a chi-squared test, followed by multiple comparisons using R version 3.3.0 [37].

## 3. Results

### 3.1. Nematodes

#### 3.1.1. Experiment 1: EPN Virulence

**Figure 1:**
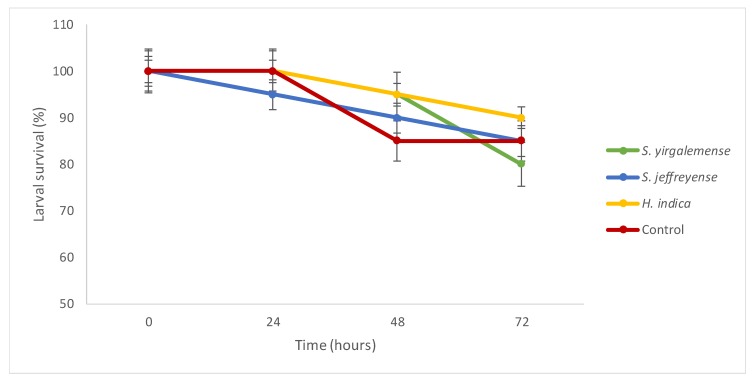
Percentage of *Cacosceles newmannii* larvae surviving infection by the three nematodes species, for both trials combined. Data is shown with standard errors of the mean (SEM).

In the first trial, none of the negative control *C. newmannii* larvae died, and three out of 10 were dead at the end of the second trial (Figure 1). All positive control (*G. mellonella* larvae) were infected and dead by the end of the trials. After 72 hours, the number of dead *C. newmannii* larvae per nematode species ranged between 2 and 4 out of 20 specimens, with 80% to 90% being alive, for both trials combined. The number of dead specimens for each nematode species did not differ from each other (*X*^2^ = 0.7843, *p*-value = 0.8532).

Dissection of the dead *C. newmannii* larvae revealed that only one larva of the first trial was infected by *S. jeffreyense* (Video S1).

#### 3.1.2. Experiment 2: Nematode Penetration

The number of nematodes found in the *C. newmannii* larvae inoculated with a high number of IJs are reported in Table 3, which ranged from 4 to 28 nematodes per insect. All larvae were still alive after the 48 hours of incubation. The penetration values associated with each nematode species were not significantly different (*t* = 0.0271, *p*-value = 0.9809).

#### 3.1.3. Experiment 3: Nematode Development in Haemolymph

**Figure 2:**
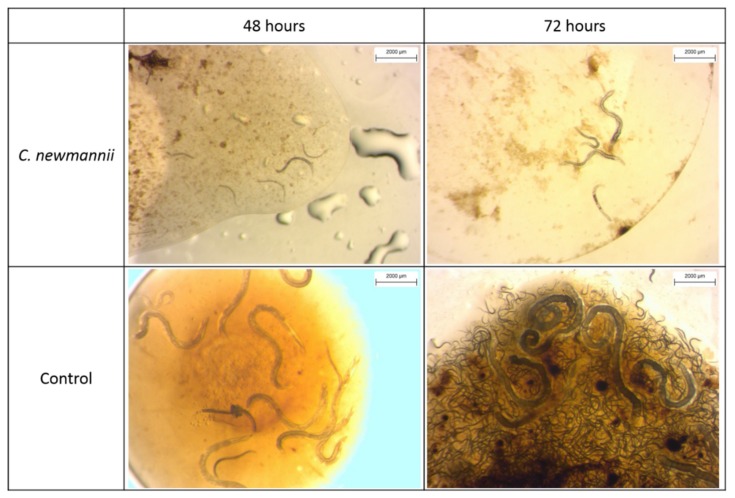
*Steinernema jeffreyense* inoculated in *Cacosceles newmannii* haemolymph **(top)** and *Galleria mellonella* haemolymph **(control, bottom)**, 48 h and 72 h after inoculation.

When a drop of haemolymph was inoculated with *S. jeffreyense* nematodes, a marked difference was observed between the control (*G. mellonella* haemolymph) and the tested *C. newmannii* haemolymph. In the control haemolymph, most inoculated IJs developed into adults, most females had been fertilized after only 48 h, and progeny was visible and abundant between 72 h and 96 h after inoculation (Table 4, Figure 2). When *C. newmannii* haemolymph was inoculated, on the other hand, most nematodes were still unrecovered (non-feeding) IJs after 48 h (Figure 2). Progeny was visible in two replicates after 96 h, and in 3 replicates after 120 h (Table 4) but were scarce and lethargic. Visual observation showed that nematodes were much more active in *G. mellonella* haemolymph than in *C. newmannii* haemolymph, and tended to be bigger. However, these observations were not formally quantified.

#### 3.1.4. Experiment 4: Virulence of Mutualistic Bacteria

Finally, when bacteria were injected into *C. newmannii* larvae, five dead specimens were reported after 48 h, the five remaining ones being relatively inactive. After 5 days, all 10 replicates were dead. Control replicates (*G. mellonella* larvae) that had been injected with bacteria all died after less than 24 h, regardless of the volume of bacterial suspension injected. Dissections showed that all internal organs were dissolved for *G. mellonella* larvae, whereas organs were still intact in *C. newmannii* larvae, indicating that the bacteria did develop in *G. mellonella* larvae, but not in *C. newmannii* larvae. The *C. newmannii* larvae injected with 2 µL of distilled water survived the treatment and were still alive after 5 days, indicating that the liquid injection itself did not alter the larval survival.

### 3.2. Experiment 5: Virulence of EPF

**Figure 3:**
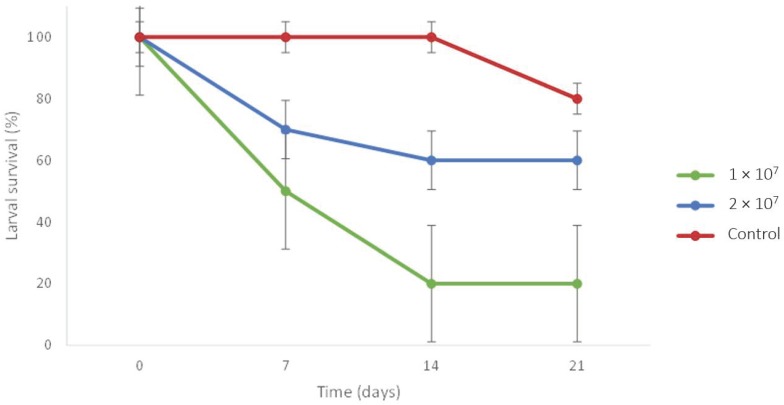
Percentage of larvae surviving infection by *M. pinghaense* at two different concentrations. Data is shown with SEM.

All positive controls were dead and showed clear sign of infection by *M. pinghaense* at the end of the first week. No death was observed among the negative *C. newmannii* control before the 3rd week of the experiment, whereas *C. newmannii* larvae in contact with the fungus started to die from the first week (Figure 3).

Overall, the mortality rates observed at each of the two concentrations and for the control treatment differed significantly (X^2^ = 7.5, *p*-value = 0.0235). Multiple comparisons however showed that only the lowest concentration (1 × 10^7^ spores per mL of water) and the control treatment significantly differed in terms of mortality of 21 days (X^2^ = 7.2, *p*-value = 0.0073). Even though the comparison was non-significant (X^2^ = 3.3, *p*-value = 0.0679), the mortality rate was always higher for the lower concentration of spores (Figure 3), and only 20% of the larvae exposed to the lowest concentration of spores (1 × 10^7^ spores per mL of water) were still alive by the end of the experiment, whereas 60% of the larvae exposed at the higher concentration (2 × 10^7^ spores per mL of water) survived. However, none of the *C. newmannii* specimens died because of the fungus itself, since none of the dead insects showed characteristic signs of mycosis.

## 4. Discussion

This study is the first to assess the virulence of EPNs and an entomopathogenic fungus in the context of biocontrol of larvae of the cerambycid *C. newmannii*. Overall, larvae of this emerging crop pest appear to be resistant to the different EPN and EPF species used in this study.

Despite the promising results that nematodes gave on other diverse insect pest species [13,43], *S. yirgalemense*, *S. jeffreyense*, and *H. indica* did not cause an increase in larval mortality compared to the negative control. Several insect species have already shown strong resistance to EPNs, and this resistance can be due to a large variety of factors, including immune defense against nematodes, or interaction with the biotic and abiotic environments [12,44]. Many of these species spend their entire larval stage in the soil, and therefore are in permanent contact with the soil environment. One hypothesis could be that these species have developed mechanisms of resistance against nematode infections that are not present in species whose larval development largely happens in a nematode-free environment, such as above ground insects. Moreover, a strong and rapid encapsulation response has been observed in *C. newmannii* larvae suggestive of a pronounced immune response.

In the case of *C. newmannii*, only *S. jeffreyense* caused larval mortality. However, only a very small number of larvae were killed (1 in 20). Given this very low pathogenicity, and despite the fact that the larvae that died did not show any abnormal signs of stress, it is questionable whether the physiological state of that single individual (stress, insect approaching moulting) might have played a role in its weak resistance to *S. jeffreyense.*

Additional experiments have been conducted to understand how the development of EPNs is limited. Even when inoculated at very high concentrations, few nematodes successfully penetrated the body of *C. newmannii* larvae, and this was true for all three EPN species studied. A barrier therefore exists at the first step of infection, which can possibly be ascribed to either the IJs were not attracted to the *C. newmannii* larvae [45], or were not able enter the insect in large numbers, because of physical or behaviour barriers [44].

In addition, once inside the *C. newmannii* larva, in the haemolymph, EPNs have difficulty developing. The time required for their full development was longer than in an insect susceptible to EPNs, and they were not able to produce abundant offspring. This rather long, or even non-existent development, is related to the apparent lack of development of *X. khoisanae* in the haemolymph of the larvae of *C. newmannii*. This bacterium is normally vectored by the IJs in the host’s body, contributes to its death by causing septicaemia, and provides a source of nutrient for the nematodes [46]. In the case of *C. newmannii*, the bacteria do not develop. This explains why IJs could survive but not grow, their main nutrient source being absent. The *C. newmannii* larvae in which the bacteria were injected died long after the *G. mellonella* control larvae, and did not show symptoms of bacterial infection. We hypothesize that even though the bacteria were not able to grow in *C. newmannii* larvae, they were still able to release toxins into their environment, hence the progressive death of the larvae studied.

EPF have shown promising results with another pest of sugarcane with a similar life cycle, *D. buqueti*, in Thailand [20,21]. The EPF species *M. pinghaense* used in this study considerably altered larval survival rate, especially for the larvae exposed to the lowest concentration of spores. However, visual examination of the dead specimens did not reveal the standard signs of fungal infection, suggesting that their death was due to another factor that we were not able to identify.

## 5. Conclusions

IPM is a holistic approach that aims at reducing pest populations’ density, while limiting pesticide use and therefore increasing environment and human health [29]. It implies the use of multiple, complementary methods, that in some cases can work synergistically (i.e., [47] for grasshoppers and locusts; [25]). Indeed, some biological control techniques benefit from being used in conjunction with other strategies.

Even though the EPN and EPF species used in this study showed a rather low pathogenicity level, infection by one EPN species (*S. jeffreyense*) and one EPF species (*M. pinghaense*, however the mechanisms involved in the death of larvae still need to be investigated) lead to the death of some larvae. Before discarding them as potential biocontrol agents as part of an IPM program, their pathogenicity needs to be investigated when coupled with other biopesticides or natural enemies. 

## Figures and Tables

**Table 1:** Weight (g) of the *Cacosceles newmannii* larvae used for experiments 1, 2, 4, 5 and as control, as an indication of the size range of the larvae used.

Experiment/Trial	Treatment	Specimen Number/Weight (g)									
		1	2	3	4	5	6	7	8	9	10
Experiment 1 trial 1	*S. yirgalemense*	0.5092	0.6696	1.1424	0.5743	1.0989	0.133	1.9325	0.9046	1.1363	0.1565
	*S. jeffreyense*	1.5117	1.1034	1.1784	0.718	0.4332	1.5098	0.4571	0.3876	0.219	0.8709
	*H. indica*	1.1406	1.2646	0.4083	0.2439	0.8227	0.3296	1.8647	1.0868	0.9111	2.0262
	Control	0.6808	1.5231	1.1688	0.2032	0.7458	0.2797	0.2515	0.516	1.401	0.8454
Experiment 1 trial 2	*S. yirgalemense*	2.2417	1.8483	1.4638	1.2731	1.2127	1.4574	1.5666	1.2842	2.0365	1.1792
	*S. jeffreyense*	1.0811	1.2079	1.0275	1.9138	0.7602	1.0263	1.754	1.8591	0.1342	1.1917
	*H. indica*	0.9668	0.5257	1.1613	1.0642	0.4061	1.1529	1.1435	0.2527	0.6853	0.4843
	Control	0.9822	0.8035	1.0267	1.7146	0.6135	0.5404	0.6147	0.5446	3.4241	1.7076
Experiment 2	*S. yirgalemense*	0.9743	1.5439	1.5915	1.3206	1.6647					
	*S. jeffreyense*	1.5141	0.7764	1.3605	1.3751	1.1906					
	*H. indica*	1.4426	0.9689	1.6716	0.8531	1.6249					
	Control	1.5206	1.3141	1.6471	0.8701	1.3683					
Experiment 4	*S. jeffreyense*	1.9546	1.3257	1.1025	1.3365	1.4679	0.9835	1.2546	0.8796	1.0257	
	control	1.163	1.2869	0.8889	0.7858	2.6022					
Experiment 5	1 × 10^7^	1.4064	1.4323	0.3762	1.9986	0.568	0.8674	0.6631	1.0008	0.5686	1.8468
	2 × 10^7^	1.9988	1.4221	1.4567	2.4397	1.0324	0.9704	0.3289	0.8421	1.3158	0.8968
	Control	1.778	2.2929	1.1989	1.1747	5.8577					

**Table 2:** List and characteristics of the *Steinernema* and *Heterorhabditis* species used.

Species Name	Strain	Habitat	Locality	GenBank Accession Number	Length of IJ (µm)	Body Width of IJ (µm)	Reference
*S. yirgalemense*	157-C	Citrus orchard	Friedenheim, Mpumalanga	EU625295	685 (570–740)	29 (24–33)	[33]
*S. jeffreyense*	J194	Guava tree	Jeffrey’s Bay, Eastern Cape	KC897093	924 (784–1043)	35 (23–43)	[34]
*H. indica*	SGS	Grapevine	Bonnievale, Western Cape	GQ377411	528 (479–573)	20 (19–22)	[35]

**Table 3:** Number of nematodes found per *Cacosceles newmannii* larva inoculated with a high concentration of IJs, for the three nematodes species used.

Specimen Number	1	2	3	4	5	Penetration Value
*Steinernema yirgalemense*	8	17	25	4	11	1.3
*Steinernema jeffreyense*	10	5	13	10	4	4.2
*Heterorhabditis indica*	14	25	28	21	9	9.7

**Table 4:** Number of females, males, IJs, and dead *Steinernema jeffreyense* 48 h after inoculation in the haemolymph, and status of the nematodes (P = progeny, E = females with eggs) 72, 96 and 120 h after inoculation. * indicate a peculiarly low level of progeny.

	**48 h**						**72 h**	**96 h**	**120 h**
***Galleria mellonella***	**Females**	**Females with Eggs**	**Males**	**Infective Juveniles**	**Dead**	**Total Inoculated**	**Status**	**Status**	**Status**
1	7	6	7	0	0	14	P	P	P
2	2	1	6	2	0	10	E	P	P
3	3	2	1	2	1	7	E	P	P
4	10	10	3	1	0	14	P	P	P
5	2	2	2	0	4	8	P	P	P
6	1	0	4	0	3	8	-	E	P
7	2	2	5	1	0	8	E	P	P
8	7	7	4	0	0	11	P	P	P
9	6	1	2	0	0	8	E	P	P
10	1	0	5	4	0	10	E	P	P
	**48 h**						**72h**	**96h**	**120h**
***Cacosceles newmannii***	**Females**	**Females with Eggs**	**Males**	**Infective Juveniles**	**Dead**	**Total Inoculated**	**Status**	**Status**	**Status**
1	0	0	1	9	0	10	-	-	-
2	0	0	6	3	0	9	-	-	-
3	4	0	5	4	0	13	-	-	-
4	0	0	2	5	0	7	-	-	-
5	4	0	2	2	0	8	E	E	P*
6	1	0	3	5	0	9	-	-	-
7	1	0	6	1	0	8	E	E	E
8	2	0	2	0	3	7	-	P*	P*
9	1	0	2	7	0	10	E	P*	P*
10	2	0	5	1	0	8	-	-	-

## Supplementary Materials

The following are available online at https://www.mdpi.com/2075-4450/10/4/117/s1, Video S1: *Steinernema jeffreyense* infection on a *Cacosceles newmannii* larva.

## References

[1] Ferreira G.W.S. (1980). The Parandrinae and the Prioninae of Southern Africa (Cerambycidae, Coleoptera).

[2] Way M.J., Conlong D.E., Rutherford R.S., Sweby D.L., Gillespie D.Y., Stranack R.A., Lagerwall G., Grobbelaar E., Perissinotto R. *Cacosceles* (*Zelogenes*) *newmannii* (Thomson) (Cerambycidae:Prioninae), a new pest in the South African sugarcane industry. Proceedings of the 90th Annual Congress of the South African Sugar Technologists Association.

[3] Javal M., Thomas S., Barton M.G., Gillespie D., Conlong D.E., Terblanche J.S. Understanding the recent invasion of *Cacosceles newmannii* (Coleoptera: Cerambycidae) into sugarcane from a thermal perspective. Proceedings of the 91st Annual Congress of the South African Sugar Technologists Association.

[4] Oyafuso A., Arakaki N., Sadoyama Y., Kishita M., Kawamura F., Ishimine M., Kinjo M., Hirai Y. (2002). Life history of the white grub *Dasylepida* sp. (Coleoptera: Scarabaeidae), a new and severe pest on sugarcane on the Miyako Islands, Okinawa. Appl. Entomol. Zool..

[5] Mukunthen N., Nirmala R. (2002). New insect pests of sugarcane in India. SugarTech.

[6] Dolinski C., Del Valle E., Stuart R.J. (2006). Virulence of entomopathogenic nematodes to larvae of the guava weevil, *Conotrachelus psidii* (Coleoptera: Curculionidae), in laboratory and greenhouse experiments. Biol. Control.

[7] Pliansinchai U., Jarnkoon V., Siengsri S., Kaenkong C., Pangma S., Weerathaworn P. Ecology and destructive behaviour of cane boring grub (*Dorysthenes buqueti* Guerin) in North Eastern Thailand. Proceedings of the XXVI Congress of the International Society of Sugar Cane Technologists.

[8] Fravel D.R. (2005). Commercialization and implementation of biocontrol. Annu. Rev. Phytopathol..

[9] Lacey L.A., Frutos R., Kaya H.K., Vail P. (2001). Insect pathogens as biological control agents: Do they have a future?. Biol. Control.

[10] Bourguet D., Guillemaud T. (2016). The hidden and external costs of pesticide use. Sustain. Agric. Rev..

[11] Peters A. (1996). The natural host range of *Steinernema* and *Heterorhabditis* spp. and their impact on insect populations. Biocontrol Sci. Technol..

[12] Labaude S., Griffin C.T. (2018). Transmission success of entomopathogenic nematodes used in pest control. Insects.

[13] Campos-Herrera R. (2015). Nematode Pathogenesis of Insects and Other Pests: Ecology and Applied Technologies for Sustainable Plant and Crop Protection.

[14] Poinar G., Gaugler R., Kaya H.K. (1990). Entomopathogenic nematodes in biological control. Taxonomy and Biology of Steinernematidae and Heterorhabditidae.

[15] Kaya H.K., Aguillera M.M., Alumai A., Choo H.Y., de la Torre M., Fodor A., Ganguly S., Hazır S., Lakatos T., Pye A. (2006). Status of entomopathogenic nematodes and their symbiotic bacteria from selected countries or regions of the world. Biol. Control.

[16] Schroeder W.J., Beavers J.B. (1987). Movement of the Entomogenous Nematodes of the Families Heterorhabditidae and Steinernematidae in Soil. J. Nematol..

[17] Poinar G.O. (1979). Nematodes for Biological Control of Insects.

[18] Simões N., Rosa J.S. (1996). Pathogenicity and host specificity of entomopathogenic nematodes. Biocontrol Sci. Technol..

[19] Ehlers R.-U. (2001). Mass production of entomopathogenic nematodes for plant protection. Appl. Microbiol. Biotechnol..

[20] Suasa-ard W., Suksen K., Kernasa O. (2012). Utilisation of the green muscardine, *Metarhizium anisopliae*, to controle the sugarcane longhorne stem borer *Dorysthenes buqueti* Guerin (Coleoptera: Cerambycidae). Int. Sugar J..

[21] Suasa-ard W., Sommartya P., Buchatian P., Puntongcum A., Chiangsin R. Effect of *Metarhizium anisopliae* on infection of sugarcane stems borer, *Dorysthenes buqueti* Guerin (Coleoptera: Cerambycidae) in laboratory. Proceedings of the 46th Kasetsart University Annual Conference.

[22] Jackson M.A., Jaronskib S.T. (2009). Production of microsclerotia of the fungal entomopathogen *Metarhizium anisoplia**e* and their potential for use as a biocontrol agent for soil-inhabiting insects. Mycol. Res..

[23] Hajek A.E., McManus M.L., Delalibera I. (2007). A review of introductions of pathogens and nematodes for classical biological control of insects and mites. Biol. Control.

[24] Marannino P., Santiago-Álvarez C., de Lillo E., Quesada-Moraga E. (2008). Evaluation of *Metarhizium anisopliae* (Metsch) Sorok. to target larvae and adults of *Capnodis tenebrionis* (L.) (Coleoptera: Buprestidae) in soil and fiber band applications. J. Invertebr. Pathol..

[25] Shah P.A., Pell J.K. (2003). Entomopathogenic fungi as biological control agents. Appl. Microbiol. Biotechnol..

[26] Malan A.P., Ferreira T., Fourie H., Spaull V.W., Jones R.K., Daneel M.S., De Waele D. (2017). Entomopathogenic nematodes. Nematology in South Africa: A View from the 21st Century.

[27] Ehlers R.-U. (1996). Current and future use of nematodes in biocontrol: Practice and commercial aspects with regard to regulatory policy issues. Biocontrol Sci. Technol..

[28] Hatting J.L., Moore S.D., Malan A.P. (2018). Microbial control of phytophagous invertebrate pests in South Africa: Current status and future prospects. J. Invertebr. Pathol..

[29] Ehler L.E. (2006). Integrated pest management (IPM): Definition, historical development and implementation, and the other IPM. Pest Manag. Sci..

[30] Van Zyl C., Malan A.P. (2015). Cost-effective culturing of *Galleria mellonella* and *Tenebrio molitor* and nematode production in various hosts. Afr. Entomol..

[31] Abaajeh A.R., Nchu F. (2015). Isolation and pathogenicity of some South African entomopathogenic fungi (Ascomycota) against eggs and larvae of *Cydia pomonella* (Lepidoptera: Tortricidae). Biocontrol Sci. Technol..

[32] Malan A.P., Nguyen K.B., Addison M.F. (2006). Entomopathogenic nematodes (Steinernematidae and Heterorhabditidae) from the southwestern parts of South Africa. Afr. Plant Prot..

[33] Malan A.P., Knoetze R., Moore S.D. (2011). Isolation and identification of entomopathogenic nematodes from citrus orchards and their biocontrol potential against false codling moth. J. Invertebr. Patholol..

[34] Malan A.P., Knoetze R., Tiedt L.R. (2016). *Steinernema jeffreyense* n. sp. (Rhabditida: Steinernematidae), new entomopathogenic nematode from South Africa. J. Helminthol..

[35] Poinar G.O., Karunakar G.K., David H. (1992). *Heterorhabditis indicus* n. sp. (Rhabditida: Nematoda) fom India: Separation of Heterorhabditis spp. by infective juveniles. Fundam. Appl. Nematol..

[36] Glazer I., Lewis E.E., Navon A., Ascher K.R.S. (2000). Bioassays for entomopathogenic nematodes. Bioassays of Entomopathogenic Microbes and Nematodes.

[37] R Core Team (2016). R: A Language and Environment for Statistical Computing.

[38] Dreyer J., Malan A.P., Dicks L.M.T. (2017). Three novel *Xenorhabdus*–*Steinernema* associations and evidence of strains of *X. khoisanae* switching between different clades. Curr. Microbiol..

[39] Ferreira T., Malan A.P. (2014). In vitro Liquid Culture of a South African Isolate of Heterorhabditis zealandica for the Control of Insect Pests. Afr. Entomol..

[40] Ekesi S., Chabi-Olaye A., Subramanian S., Borgemeister C. (2011). Horticultural pest management and the African economy: Successes, challenges and opportunities in a changing global environment. Acta Hortic..

[41] Brunner-Mendoza C., Del Rocío Reyes-Montes M., Moonjely S., Bidochka M.J., Toriello C. (2019). A review on the genus *Metarhizium* as an entomopathogenic microbial biocontrol agent with emphasis on its use and utility in Mexico. Biocontrol Sci. Technol..

[42] Mathulwe L.L. (2019). Control of the Woolly Apple Aphid, *Eriosoma lanigerum* (Hausmann) (Hemiptera: Aphididae), Using Entomopathogenic Fungi. Master’s Thesis.

[43] Malan A.P., Hatting J., Campos-Herrera R. (2015). Entomopathogenic Nematode Exploitation: Case Studies in Laboratory and Field Applications from South Africa. Sustainability in Plant and Crop Protection: Ecology and Applied Technologies for Sustainable Plant and Crop Protection.

[44] Narayanan K. (2006). Insect defence: Its impact on microbial control of insect pests. Curr. Sci..

[45] Baiocchi T., Lee G., Chloe D.-H., Dillman A.R. (2017). Host seeking parasitic nematodes use specific odors to assess host resources. Sci. Rep..

[46] Banu J.G., Gannayane I., Meena K.S., Abd-Elgawad M., Askary T.H., Coupland J. (2017). Entomopathogenic nematodes: General biology and behaviour. Biocontrol Agents Entomopathogenic and Slug Parasitic Nematodes.

[47] Lomer C.J., Bateman R.P., Johnson D.L., Langewald J., Thomas M. (2001). Biological control of locusts and grasshoppers. Annu. Rev. Entomol..

